# An MNase-ChIP-Seq Protocol to Profile Histone Modifications at a DNA Break in Yeast

**DOI:** 10.3390/mps9020042

**Published:** 2026-03-07

**Authors:** Elena Di Nisio, Chiara Frigerio, Valerio Licursi, Sara Castelli, Benedetta Caraba, Rodolfo Negri, Michela Clerici

**Affiliations:** 1 Department of Biology and Biotechnologies “C. Darwin”, Sapienza University of Rome, 00185 Rome, Italy; elena.dinisio@uniroma1.it (E.D.N.); benedetta.caraba@uniroma1.it (B.C.); rodolfo.negri@uniroma1.it (R.N.); 2 Department of Biotechnology and Biosciences, University of Milano-Bicocca, 20126 Milan, Italy; c.frigerio32@campus.unimib.it (C.F.); s.castelli30@campus.unimib.it (S.C.); 3 Institute of Molecular Biology and Pathology (IBPM), National Research Council (CNR) of Italy, 00185 Rome, Italy

**Keywords:** chromatin immunoprecipitation and DNA sequencing (ChIP-seq), micrococcal nuclease, histone post-translational modification, DNA double-strand break, *Saccharomyces cerevisiae*

## Abstract

Eukaryotic DNA is wrapped around octamers of four core histones, forming nucleosomes. Histone post-translational modifications (PTMs) influence chromatin structure and the recruitment of regulatory factors, thereby affecting gene expression and DNA repair, including the response to DNA double-strand breaks (DSBs). Here, we describe a robust chromatin immunoprecipitation protocol combined with micrococcal nuclease digestion and DNA sequencing (MNase-ChIP-seq) to map histone modifications and their genome-wide distribution after the induction of a single DSB by the HO endonuclease in *Saccharomyces cerevisiae*. We validate the method by detecting changes in histone H3 methylation following *HO* transcriptional activation and DSB induction. This protocol enables reliable analysis of histone PTMs across mutant strains or stress conditions, supporting studies of chromatin dynamics in yeast.

## 1. Introduction

The DNA double-strand break (DSB) is one of the most dangerous lesions that DNA can incur, as it involves the cleavage of both strands. Cells exhibit a very limited tolerance to DNA DSBs. In yeast haploid cells, a single unrepaired DSB is highly cytotoxic and leads to prolonged cell-cycle arrest and cell death [1]. Yeast cells also lose viability when few (1–10) DSBs are generated concomitantly, depending on ploidy and the repair proficiency of the cells [2,3]. Similarly, in mammalian cells with defects in DNA repair pathways, even just a few DSBs—or even just one—can trigger apoptosis and cell death [4,5]. Cells counteract this highly deleterious damage by activating a coordinated response that detects the DSB, signals its presence and promotes its repair, thereby safeguarding genome integrity [6]. Two main systems can repair DSBs: non-homologous end joining (NHEJ) and homologous recombination (HR). NHEJ relies on DNA ligase activity to directly ligate broken DNA ends, while HR uses a homologous DNA sequence as a template for homology-driven repair. The choice of the DSB repair pathway depends on which proteins bind DSB ends and how their activities are regulated [7,8]. In particular, nucleolytic degradation of DNA ends in the 5′-to-3′ direction is essential to generate the 3′-ended single-stranded DNA (ssDNA) tails that promote homology search and recombination [7,9].

DNA DSBs occur within the context of chromatin and the DSB response is modulated by chromatin modifications [8]. In nucleosomes, the DNA is wrapped around octamers of four core histones H2A, H2B, H3, and H4. Histone tails protrude from the nucleosomes and are subjected to a vast array of post-translational modifications (PTMs), including phosphorylation, acetylation, methylation, and ubiquitylation [10,11,12]. They affect chromatin structure either by modifying histone–histone or histone–DNA interactions, or by providing binding sites for other chromatin remodeling factors [13,14]. Although several histone PTMs have been characterized for their role in promoting different repair pathways, a comprehensive view of the complex interplays among histone PTMs and DSB repair factors is still elusive.

Chromatin Immunoprecipitation (ChIP) and Cleavage Under Targets and Release Using Nuclease (CUT&RUN) are currently the most widely used methods for investigating the genome-wide distribution of chromatin-bound proteins and histone PTMs [15,16].

In ChIP, cells are treated with formaldehyde to induce crosslinking between proteins and DNA. Then, chromatin is extracted and fragmented into a manageable size (~150–300 base pairs) by sonication or enzymatic treatments, according to the purpose of the experiment [16]. This step is followed by immunoprecipitation of the target protein with specific antibodies and identification of DNA sequences in the immunoprecipitated sample through real-time PCR or sequencing of the copurifying DNA fragments [15,17]. For the enzymatic chromatin fragmentation, micrococcal nuclease (MNase) is commonly used. This enzyme is an endo- and exo-nuclease that preferentially digests the naked DNA between nucleosomes, thus releasing the nucleosomes from chromatin and enriching the nucleosome-protected DNA fragments [18]. Although MNase exhibits intrinsic sequence preferences for DNA cleavage [19], it has been widely and effectively used as a probe for DNA accessibility, because nucleosomes and other DNA-binding proteins efficiently protect their associated DNA from MNase digestion. Importantly, MNase’s sequence bias is strongly constrained by DNA accessibility: cleavage at inward-facing nucleosomal sites primarily reflects local accessibility rather than intrinsic sequence preference, indicating that protein binding can override sequence biases [20]. Fragmentation with MNase is particularly useful for studying histone PTMs and generates high-resolution data while eliminating signal artifacts caused by crosslinking [16]. Conversely, sonication generates fragments that are less uniform in size, and may be preferable for ChIP of non-histone proteins, such as transcription factors, which tend to bind linker DNA [16,17].

In the CUT&RUN assay, a specific antibody recognizes the target protein in permeabilized but otherwise intact cells. DNA fragmentation is then achieved by MNase linked to protein A or protein G. The nuclease cleaves the linker DNA flanking the nucleosome bound by the antibody, thus releasing the DNA fragment wrapped around that nucleosome. Because this method does not require crosslinking and DNA is not fragmented before immunoprecipitation, protein–DNA interactions are likely maintained in their natural state. Together with the CUT&TAG assay (Cleavage Under Targets and Tagmentation), CUT&RUN has become widely used in recent years due to the low amounts of starting cells required compared with ChIP-based approaches [21,22]. This advantage, however, is less critical when working with yeast cells, because the biological material is typically abundant.

Here, we describe a ChIP-based protocol (MNase-ChIP-seq), in which chromatin is fragmented by MNase digestion and subsequently analyzed by Next-Generation DNA Sequencing (NGS) to map histone PTMs and their genome-wide distribution after the induction of a single DSB at a defined locus in *Saccharomyces cerevisiae*. Similar strategies have been already developed [23] and successfully applied in yeast [24], where the abundance of biological material makes this approach particularly convenient for profiling chromatin-associated proteins.

Chromatin modifications and DNA repair are highly dynamic processes at a DNA break, where histone PTMs can rapidly appear and disappear depending on the kinetics of DSB processing and repair [25]. Taking these dynamics into account, the persistence of the DSB and its unique location provide an advantage for the study of histone PTMs and their evolution over time. This requirement is met by the yeast haploid strain JKM139 [1], where a single DSB is generated synchronously in most of the cells of a population by an inducible system, and persists for a long time. This strain carries a galactose-inducible version of the homothallic switching endonuclease HO integrated in the genome (*ade3::GAL-HO*), which creates a DSB at the *MATa* locus on chromosome III by recognizing a unique recognition sequence. This HO-induced DSB cannot be repaired by HR, since the *HML* and *HMR* homologous regions have been deleted [1]. A ChIP-based approach is likely more suitable than CUT&RUN for studying histone PTMs in this system, where the cell number is not limiting. In ChIP, chromatin is fragmented by MNase after crosslinking and cell lysis, and before immunoprecipitation. Conversely, in CUT&RUN chromatin cleavage is performed only after the binding of the antibody to chromatin into permeabilized cells. This difference is particularly relevant for yeast cells, whose permeabilization requires enzymatic treatments (i.e., Zymolyase digestion), due to the presence of the cell wall. This step may only be partially efficient after crosslinking, potentially increasing the background in the CUT&RUN assay. Moreover, Zymolyase preparations are known to contain protease activities, which could further interfere with the assay and compromise data quality.

The use of the DSB-inducible system and the availability of different techniques to analyze events at the HO-induced DSB offer several opportunities to define the dynamics occurring at the DSB: (i) histone PTMs and the generation of ssDNA at the HO-induced DSB [26] can be monitored in the same experiment, thus allowing the correlation of histone PTMs with DSB processing; (ii) similarly, the recruitment of repair or checkpoint factors at DSB can be monitored by ChIP-qPCR, allowing the correlation of histone PTMs with the activation of the DNA damage response and repair pathways; (iii) as *MATa* cells can be synchronized in G1 with the α-factor pheromone, these analyses can be carried out in different cell cycle stages, enabling the investigation of cell cycle-specific histone PTMs. Furthermore, this protocol is robust and versatile and can be applied to other genetic backgrounds, in which DSBs are generated in different genomic loci or by different nucleases, and can be repaired. Finally, it enables reliable analyses of histone PTMs across mutant strains, supporting studies of chromatin dynamics in yeast.

## 2. Experimental Design

**Figure 1:**
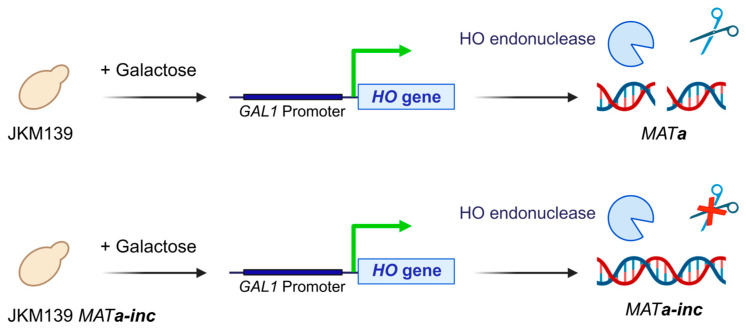
HO induction and DSB formation. Galactose is added to JKM139 and JKM139 *MATa-inc* cells growing in YEPR medium to induce the expression of the HO endonuclease by the *GAL1* promoter. HO creates a DSB at the *MATa* locus of JKM139 cells but not in JKM139 *MATa-inc* cells. Symbols: green arrow, transcriptional activation; scissor, cutting by HO endonuclease; scissor with red X, cutting blocked at the locus *MATa-inc*. Created using BioRender.com.

We describe a MNase-ChIP-seq experiment to define the panel of histone PTMs in response to the generation of an unrepairable DSB. The yeast strain JKM139 is used to induce an unrepairable DSB at the *MATa* locus by the galactose-inducible expression of HO [1]. To confirm that variations in histone PTMs are specifically induced by DSB formation, the control isogenic strain JKM139 *MATa-inc*, carrying a mutation in the HO recognition sequence (*MATa-inc*) that prevents cleavage at the *MATa* locus, is used [27] (Figure 1).

JKM139 and the control JKM139 *MATa-inc* strains are grown in raffinose-containing rich medium and galactose is added to exponentially growing cells at time 0 to induce HO expression. Samples are collected at time 0 and at different time points after galactose addition to determine HO expression and the efficiency of DSB formation, and to perform MNase-ChIP-seq. qPCR-based assays are used to confirm that both strains express HO and that the enzyme cuts the *MATa* locus only in the JKM139 cells. For ChIP analysis, chromatin is extracted from the cells after crosslinking with formaldehyde and quenching with glycine. MNase digestion of isolated chromatin is performed to obtain mainly single- and di-nucleosome fragments. Parallel immunoprecipitations from the same sample are then carried out using either an antibody that recognizes a specific histone PTM (e.g., H3K79me1) or an antibody against total histone (e.g., H3). If desired, an optional IgG negative control may be included, using IgG of the same isotype as the primary antibodies. Finally, purified DNA from immunoprecipitated and input samples is analyzed by NGS, and the obtained reads are processed using a well-established, open access bioinformatic pipeline.

### 2.1. Materials

*Commonplace materials are not identified with a supplier or catalogue number.
**In most cases, a functionally equivalent alternative material may be used.
Buffer preparation is described in “Reagents setup” (Section 5).

- 50 mL sterile conical tubes (EuroClone, Pero (MI), Italy, ET5050B).
- 15 mL sterile conical tubes (EuroClone, Pero (MI), Italy, ET5015B).
- 1.5 mL RNase/DNase-free tubes (Eppendorf, Hamburg, Germany, Cat. No. 0030120086).
- 1.5 mL DNA LoBind Tubes (Eppendorf, Hamburg, Germany, Cat. No. 0030108051).
- 1.5 mL Protein LoBind Tubes (Eppendorf, Hamburg, Germany, Cat. No. 0030108116).
- 1.5 mL screw cap tubes (VWR, Radnor, PA, USA, Cat. No. 525-0646).
- Glass beads acid-washed (Sigma-Aldrich, Darmstadt, Germany, Cat. No. G8772).
- Needles 21 Gauge (Nipro, Osaka, Japan, Cat. No. HN2138ET).
- UltraPure DNase/RNase-Free Distilled Water (Invitrogen, Thermo Fisher Scientific, Inc., Waltham, MA, USA, Cat. No. 10977035).
- Bacto peptone (FORMEDIUM Ltd., King’s lynn, England, Cat. No. PEP03).
- Yeast extract (FORMEDIUM, King’s lynn, England, Cat. No. YEA03).
- D-(+)-glucose monohydrate (Sigma-Aldrich, Darmstadt, Germany, Cat. No. 49159).
- Adenine hemisulfate salt (Sigma-Aldrich, Darmstadt, Germany, Cat. No. A9126).
- D-(+)-Raffinose (Sigma-Aldrich, Darmstadt, Germany, Cat. No. 83400).
- D-(+)-Galactose (Sigma-Aldrich, Darmstadt, Germany, Cat. No. 48260).
- Phenol–chloroform–isoamyl alcohol (PCI) 25:24:1 (Sigma-Aldrich, Darmstadt, Germany, Cat. No. 77617).
- Ethanol 99% (EtOH) (Sigma-Aldrich, Darmstadt, Germany, Cat. No. 1.00983.2511).
- DNA-free kit (Invitrogen, Thermo Fisher Scientific, Inc., Waltham, MA, USA Cat. No. AM1906).
- SensiFAST cDNA Synthesis Kit (Meridian Bioscience, Inc., Cincinnati, OH, USA, Cat. No. BIO-65053).
- SensiFAST™ SYBR^®^ Hi-ROX Kit (Meridian Bioscience, Inc., Cincinnati, OH, USA, Cat. No. BIO-92020).
- Sso Fast EvaGreen supermix (Bio-Rad, Hercules, CA, USA, Cat. No. 1725204).
- D-Sorbitol (Sigma-Aldrich, Darmstadt, Germany, Cat. No. S1876).
- Thylenediaminetetraacetic acid (EDTA) (Sigma-Aldrich, Darmstadt, Germany, Cat. No. E9884).
- Zymolyase 20T^®^ from *Arthrobacter luteus* (Nacalai Tesque, Kyoto Japan Cat. No. 07663-91).
- β−mercaptoethanol (Sigma-Aldrich, Darmstadt, Germany, Cat. No. 444203).
- Tris-HCl pH 7.5 1 M, 1000 mL (Invitrogen, ThermoScientific, Waltham, MA, USA, Cat. No. 15567027).
- UltraPure SDS Solution, 10% (Invitrogen, ThermoScientific, Waltham, MA, USA, Cat. No. 15553027).
- Trizma^®^base (Sigma-Aldrich, Darmstadt, Germany, Cat. No. T6066).
- Sodium hydroxide (10 N) (Sigma-Aldrich, Darmstadt, Germany, Cat. No. 72068-100 ML).
- Potassium Acetate (Kac) (Sigma-Aldrich, Darmstadt, Germany, Cat. No. 236497).
- Sodium chloride, 99.85%, for molecular biology, DNase, RNase and Protease-free (NaCl) (Sigma-Aldrich, Darmstadt, Germany, Cat. No. 327300010).
- PureLink RNase A (20 mg/mL) (ThermoScientific, Waltham, MA, USA, Cat. No. 12091021).
- Formaldehyde 37% (Sigma-Aldrich, Darmstadt, Germany, Cat. No. F8775).
- Glycine, ≥99%, Molecular Biology-Grade, Ultrapure (ThermoScientific, Waltham, MA, USA, Cat. No. J16407.36).
- Phosphate-Buffered Saline (PBS) (Sigma-Aldrich, Darmstadt, Germany, Cat. No. P4417).
- 4-(2-hydroxyethyl)-1-piperazineethanesulfonic acid (HEPES) (Sigma-Aldrich, Darmstadt, Germany, Cat. No. H4034).
- Sodium deoxycholate (NadEox) (Sigma-Aldrich, Darmstadt, Germany, Cat. No. 30970).
- Octylphenoxy poly(ethyleneoxy)ethanol, branched (IGEPAL) (Sigma-Aldrich, Darmstadt, Germany, Cat. No. I8896).
- Phenylmethanesulfonyl fluoride (PMSF) (Sigma-Aldrich, Darmstadt, Germany, Cat. No. 78830).
- Protease Inhibitor Cocktail Tablets, cOmplete Mini, (Sigma-Aldrich, Darmstadt, Germany, Cat. No. 11836153001 Roche).
- Halt protease inhibitor cocktail, EDTA-free (100×), 1 mL (ThermoScientific, Waltham, MA, USA, Cat. No. 87785).
- Calcium chloride dihydrate (CaCl_2_) (Sigma-Aldrich, Darmstadt, Germany, Cat. No. 223506).
- Benzamidine Hydrochloride Hydrate (Sigma-Aldrich, Darmstadt, Germany, Cat. No. B6506-5G).
- Aprotinin (ThermoScientific, Waltham, MA, USA, Cat. no. 78432-25MG).
- 2-(Aminoethyl)benzenesulfonyl fluoride hydrochloride (AEBSF) (ThermoScientific, Waltham, MA, USA, Cat. No.78431-100MG).
- Micrococcal Nuclease (MNase) (ThermoScientific, Waltham, MA, USA, Cat. No. 88216).
- Ethylenediaminetetraacetic acid (EDTA), 0.5 M Solution, Molecular Biology-Grade, Ultrapure (ThermoScientific, Waltham, MA, USA, Cat. No. J15694.AE).
- Proteinase K (Recombinant), PCR Grade (ThermoScientific, Waltham, MA, USA, Cat. No. EO0491).
- Anti-H3K79me1 (GeneTex, Irvine, CA, USA, Cat. No. GTX60353).
- Anti-H3 (Abcam, Cambridge, UK, Cat. No. ab1791).
- Pierce™ ChIP-grade Protein A/G Magnetic Beads (ThermoScientific, Waltham, MA, USA, Cat. o. 26162).
- Lithium Chloride (LiCl) 7.5 M solution (ThermoScientific, Waltham, MA, USA, Cat. No. AM9480).
- Sodium hydrogen carbonate, 99.7+%, ACS reagent (NaHCO_3_) (ThermoScientific, Waltham, MA, USA, Cat. No. 424270250).
- DNA purification kit (Qiagen, Hilden, Germany, Cat. No. 28106).
- DNA loading dye (New England BioLabs, Ipswich, MA, USA, Cat. No. B7024S).
- Tris EDTA buffer, for molecular biology, DNase, RNase, Protease-free ready to use, pH 8.0 (ThermoScientific, Waltham, MA, USA, Cat. No. 327345000).
- DNA ladder (EuroClone, Pero (MI), Italy, Cat. No. EMR816100).
- Agarose (EuroClone, Pero (MI), Italy, Cat. No. EMR920500).
- 2−propanol anhydrous, 99.5% (Sigma-Aldrich, Darmstadt, Germany, Cat. No. I9030).
- Ovation^®^ Ultralow V2 DNA-Seq Library Preparation Kit (Tecan, Redwood City, CA, USA).

### 2.2. Equipment

- Dubnoff water bath.
- Spectrophotometer.
- Shaker.
- Refrigerated centrifuge.
- Refrigerated microcentrifuge.
- Bead beater.
- Thermal mixer.
- Nutator.
- Vortex.
- Magnetic racks.
- Equipment for gel electrophoresis.
−80 °C freezer.
- Nanodrop.
- Qubit 2.0 Fluorometer (Invitrogen, Carlsbad, CA, USA).
- HS DNA assay Bioanalyzer (Agilent, Santa Clara, CA, USA).
- NovaSeqX Plus platform (Illumina, San Diego, CA, USA).

## 3. Procedure

**Figure 2:**
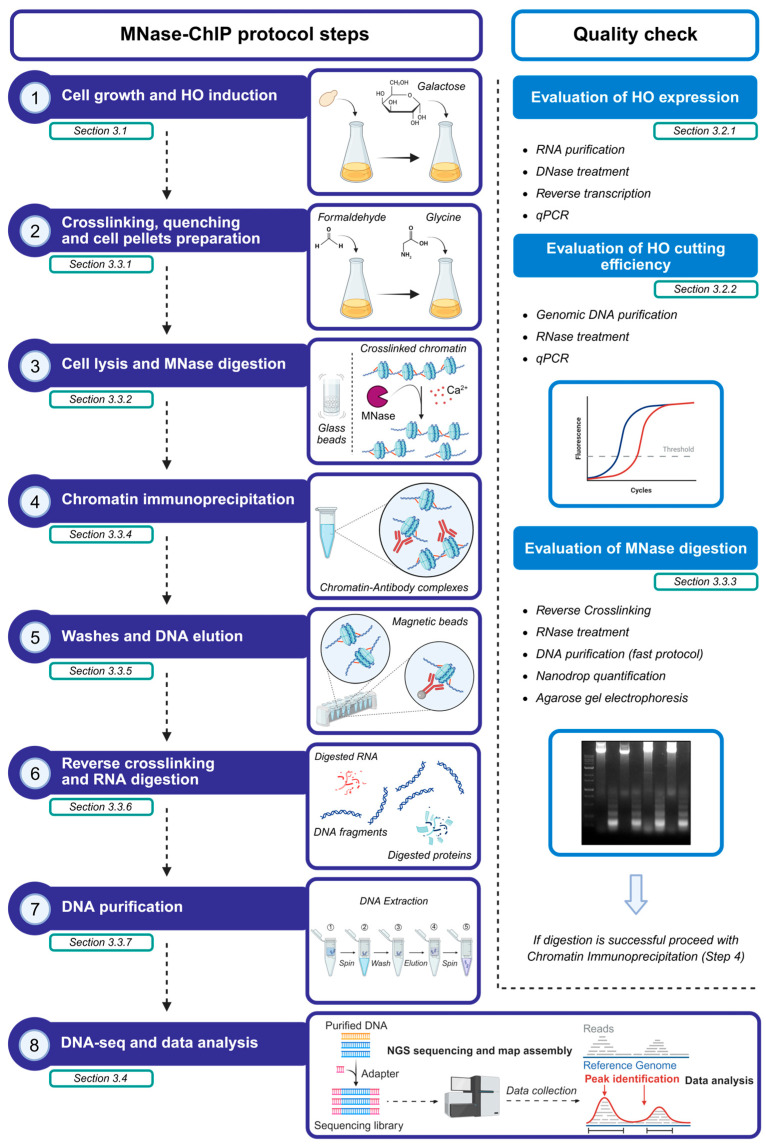
Schematic workflow of MNase-ChIP-seq protocol and quality checks. The critical steps of the protocol are summarized in the left column, and the quality checks that must be performed before proceeding with the chromatin immunoprecipitation are described in the right column. The respective in-depth sections in the manuscript are referenced. The image was created using BioRender.com.

To highlight critical steps and quality checks (Figure 2), we divide the protocol into four main parts: (i) yeast cell growth and DSB induction; (ii) evaluation of HO expression and HO cut efficiency; (iii) chromatin immunoprecipitation; (iv) DNA sequencing and data analysis.

### 3.1. Yeast Cells Growth and DSB Induction

**Figure 3:**
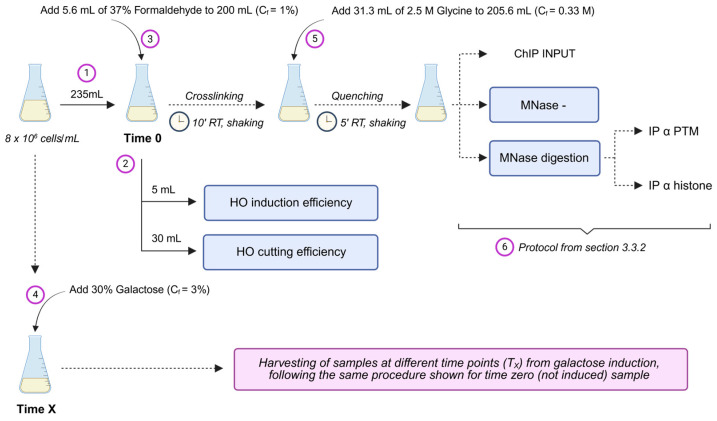
Schematic workflow of sample harvesting. The volume shown in the figure is representative of two chromatin immunoprecipitation reactions. One has an antibody directed towards a specific post-translational modification (PTM); the other has an antibody towards the total histone whose modification is to be investigated. Dashed arrows indicate successive steps in the protocol; solid arrows indicate addition of reagents or sample collection. The image was created using BioRender.com.

1. Inoculate cells in 50–100 mL YEPD medium.
2. Grow the cell cultures for 6–8 h at 25 °C in a shaking Dubnoff water bath until the exponentially growing cells reach a density of 10^4^–10^6^ cells/mL. Cell density can be determined using a Coulter counter instrument, by evaluating the number of cell units in a fixed volume, or by measuring the optical density at 600 nm (OD_600_) of the yeast culture using a spectrophotometer, considering that 0.1 OD_600_ corresponds to a culture containing approximately 1.66 × 10^6^ cells/mL.
3. Spin down the cells and wash them with YEPR medium to remove glucose. Resuspend the cells in an equal or larger volume (depending on the number of samples to collect after HO induction) of YEPR medium. For each time point, consider having 50 mL of culture for each ChIP reaction, 50 mL for the control of MNase digestion, 50 mL for the undigested control, 5 mL for RNA extraction and evaluation of HO expression, and 30 mL for DNA extraction and evaluation of HO cut efficiency. Be sure to transfer enough cells into YEPR medium to reach a density of at least 6 × 10^6^ cells/mL the next day.
4. Grow the cell culture at 25 °C for 12–16 h.
5. Dilute the culture to a concentration of 8 × 10^6^ cells/mL in your desired volume.
6. Draw the samples for the uninduced control (Time 0): 50 mL for each ChIP reaction, plus 135 mL for all the downstream quality checks (refer to step 3 above and Figure 2). Set aside 35 mL of all this volume withdrawn at time zero for the quality checks of HO induction (Section 3.2.1) and HO cut efficiency (Section 3.2.2), and proceed with crosslinking, quenching, and cell pellet preparation (Section 3.3.1) from the remaining samples collected (Figure 3). Add galactose from a 30% solution to a 3% final concentration to the remaining cell culture to induce HO expression.
7. Grow the cell culture at 25 °C and harvest the samples at the desired time points after galactose addition, as described for uninduced samples during step 6 (Figure 3).

Note: The only quality check to perform following the crosslinking and quenching steps is the control of chromatin digestion after MNase treatment, as described in Section 3.3.3.

### 3.2. Evaluation of HO Expression and DSB Formation

#### 3.2.1. Evaluation of HO Expression

From the uninduced culture (Time 0) and each experimental time point following HO induction, harvest a portion of the cell culture for RNA purification (Figure 3) and the following RT-qPCR analyses, as follows:

1. Place 5 mL of cell culture (OD_600_ = 0.5) in a 15 mL sterile tube.
2. Centrifuge at 2500 × *g* and 4 °C for 5 min, then discard the supernatant.
3. Resuspend the cell pellet in 5 mL of cold RNase-free water.
4. Centrifuge at 2500 × *g* at 4 °C for 5 min and discard the supernatant.
5. Resuspend the pellet in 200 μL of lysis buffer.
6. Transfer the volume in a 1.5 mL RNase-free tube with sterile 0.3 g micro glass beads
7. Add 200 μL of phenol–chloroform–isoamyl alcohol (PCI), then lyse by vortexing for 2 min.
8. Add another 300 μL of lysis buffer and 300 μL of PCI, vortex briefly.
9. Centrifuge at 10,000 × *g* for 5 min at 4 °C, then collect the supernatant in a new RNase-free 1.5 mL tube.
10. Proceed with the RNA precipitation by adding 3 volumes of ice-cold 99% EtOH, then mix by inverting few times.
11. Incubate the sample at −20 °C for 30 min.
12. Centrifuge the sample at 10,000 × *g* for 15 min at 4 °C, then discard the supernatant.
13. Wash the pellet with 100 μL of ice-cold 70% EtOH (do not disturb the pellet).
14. Centrifuge at 5000 × *g*, 4 °C for 1 min and remove any supernatant left.
15. Dry out the RNA pellet in a chemical hood by keeping the tube upside down.

Note: Do not exceed 10 min for drying; to fully dry out the sample, use sterile 3 mm paper strips but avoid disturbing the RNA pellet.

16. Resuspend the precipitated nucleic acids in 15 μL of RNase-free water.
17. Remove any genomic contaminants by treating the samples with DNase I (DNA-free kit, Invitrogen, Thermo Fisher Scientific, Inc., Waltham, MA, USA).

Note: Alternative kits can be used for genomic DNA removal.

18. Measure the RNA concentration using a nanodrop instrument.
19. Check the integrity of RNA via electrophoresis on 1% agarose gel in Tris-Borate-EDTA (TBE) buffer 1×.
20. Reverse-transcribe the RNA into cDNA using the SensiFAST cDNA Synthesis Kit (Meridian Bioscience, Inc., Cincinnati, OH, USA), following the manufacturer’s instructions.

Note: Alternative kits can be used for reverse transcription.

21. Proceed with qPCR analysis to determine the expression of the HO endonuclease, using the primer pairs listed in Table 1. Run the qPCR on a CFX Connect Real-Time System (Bio-Rad), a StepOnePlus (Applied Biosystems), or a comparable instrument using the SsoFast EvaGreen Supermix (Bio-Rad), with the following cycling program: 98 °C for 2 min, followed by 45 cycles of 98 °C for 5 s and 60 °C for 10 s. Other reagents can be used according to the manufacturer’s datasheet. For example, using the sensi FAST SYBRGreen Hi-ROX mix (Bioline, Meridian), use the following cycling program: 95 °C for 2 min, followed by 40 cycles of 95 °C for 5 s and 60 °C for 15 s.

#### 3.2.2. Evaluation of HO Cut Efficiency

From Time 0 sample and from each experimental time point following HO induction, harvest a portion of the cell culture for genomic DNA extraction and qPCR analyses, as follows:

1. Pellet 30 mL of the cell culture by spinning 3 min at 1600× *g* in 50 mL tubes.
2. Wash the cells in 1 mL spheroplasting solution and transfer the samples to 1.5 mL microcentrifuge tubes.
3. Centrifuge at 900× *g* for 3 min and completely remove supernatant with a tip.

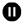
 PAUSE STEP. Store cell pellets at −20 °C until further processing; otherwise, proceed with spheroplasting preparation. Resuspend the cell pellets in 400 μL spheroplasting solution + 14 mM β−mercaptoethanol.

**Figure 4:**
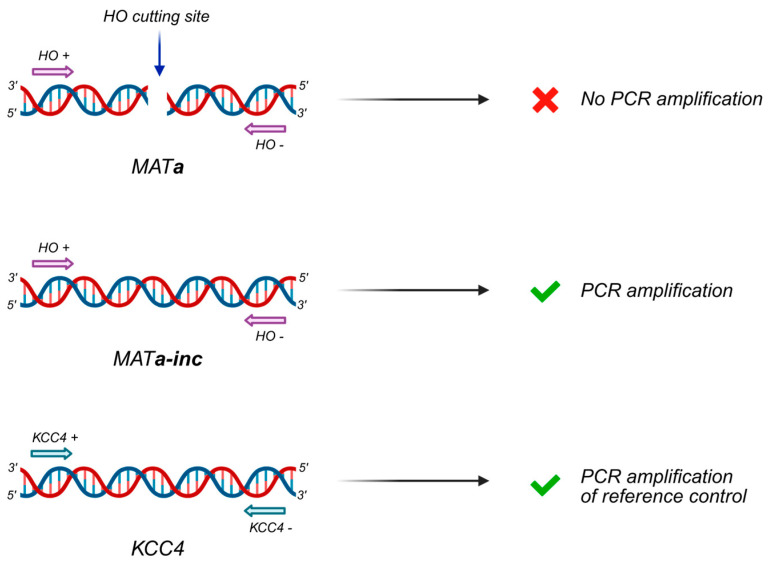
Schematic representation of the PCR assay for the evaluation of HO endonuclease cut efficiency. The image was created using BioRender.com.

4. Add 100 μL Zymolyase solution to each sample and invert the tube 4–6 times. Incubate the samples at 37 °C for 30 min.
5. Centrifuge at 18,000× *g* for 1 min and carefully remove supernatant with a tip.
6. Gently resuspend spheroplasts in 400 μL 1× TE (do not vortex).
7. Add 90 μL lysis solution (prepared just before use). Immediately shake the tube vigorously (a foam should form) and incubate the samples 30 min in a water-bath at 65 °C.
8. Add 80 μL 5 M potassium acetate and mix by inverting the tube several times. Place the tubes on ice for approximately 1 h.
9. Centrifuge at 18,000× *g* for 20 min at 4 °C. Transfer the supernatant with nucleic acids to new 1.5 mL tubes. Discard the pellets.
10. Add 1 mL ice-cold 99% ethanol and mix by inverting the tube several times. A white cloudy precipitate should form. Keep at −80 °C for 30 min to facilitate precipitation.
11. Centrifuge at 18,000× *g* for 10 min at 4 °C and remove the supernatant.
12. Wash the pellet with 1 mL ice-cold 70% ethanol and immediately discard the ethanol.
13. Air-dry the pellet until it appears glassy.
14. Add 500 μL 1× TE. Let tubes sit for 30 min at room temperature, then gently dissolve the pellet (do not vortex).
15. When all the pellets are completely dissolved and the solutions appear clear and transparent, add 2.5 μL RNase solution (10 mg/mL) to each sample and incubate 1 h at 37 °C.
16. Add 500 μL 2-propanol and invert the tube several times. A white DNA “clew” should form. Keep at −80 °C for 30 min or 12–16 h to facilitate precipitation.
17. Centrifuge at 18,000× *g* for 15–30 min at 4 °C and remove the supernatant.
18. Chromatin Immunoprecipitation 1 mL ice-cold 70% ethanol and immediately discard the ethanol.
19. Air-dry until the pellet appears glassy.
20. Add 30 μL 1× TE. Let tubes sit for 30 min at room temperature, then gently dissolve the DNA pellet (do not vortex).
21. Perform qPCR with oligonucleotides HO+ and HO- (Table 2), which anneal to opposite sides with respect to the HO cutting sequence or with KCC4+ and KCC4- primers (Table 2) that anneal to a control uncut locus (Figure 4). Run the qPCR on a CFX Connect Real-Time System (Bio-Rad), a StepOnePlus (Applied Biosystems), or a comparable instrument using the SsoFast EvaGreen Supermix (Bio-Rad), with the following cycling program: 98 °C for 2 min, followed by 45 cycles of 98 °C for 5 s and 60 °C for 10 s. Other reagents can be used according to the manufacturer’s datasheet. For example, using the sensi FAST SYBRGreen Hi-ROX mix (Bioline, Meridian), use the following cycling program: 95 °C for 2 min, followed by 40 cycles of 95 °C for 5 s and 60 °C for 15 s.
22. Determine the efficiency of DSB formation. First, Ct values of the HO amplicon are normalized to those from the KCC4 control amplicon for each time point. The obtained values after galactose addition are normalized to those obtained before galactose addition to calculate the percentage of HO-cut formation.

### 3.3. Chromatin Immunoprecipitation

#### 3.3.1. Crosslinking, Quenching and Cell Pellet Preparation

1. Transfer 235 mL of yeast culture into a glass flask and draw 35 mL before the crosslinking reaction for the quality checks (Figure 3).
2. Add 5.6 mL of 37% formaldehyde to the remaining volume for a final concentration of 1% formaldehyde. To ensure proper mixing of the culture and an efficient crosslinking reaction, a 1:5 ratio between the culture volume and the total flask volume should be maintained. Perform this step in a fume hood.

Note: It is preferable to use formaldehyde that is less than 1 month old. We suggest purchasing small quantities and using them within a short time, avoiding repeated opening/closing of the bottle, because the oxygen exposure induces the polymerization of formaldehyde, which will reduce the crosslinking efficiency.

3. Transfer the flask to a shaker at 25 °C (or room temperature) and shake it at 50 rpm for 10 min.

Note: The timing of formaldehyde crosslinking is crucial, since the extent of crosslinking strongly affects the following MNase digestion efficiency. Therefore, start the cultures in such a way that few samples are ready at the same time.

4. Add 31.3 mL of 2.5 M glycine for a final concentration of 0.33 M glycine and continue shaking at 50 rpm at room temperature for 5 min to quench the formaldehyde.
5. Split each culture over 4× a 50 mL conical tube and centrifuge at 2500× *g* for 5 min at 4 °C. Discard the supernatants.
6. Repeat step 5 using the remaining sample in the same collection tubes. Discard the supernatants.

Note: Keep the sample on ice during the centrifugations and try to be as quick as possible.

7. Wash the cell pellets by resuspending them in 40 mL of cold, sterile PBS.
8. Centrifuge at 2500× *g* for 5 min at 4 °C. Discard the supernatant.
9. Wash pellets again by resuspending the first one in 40 mL of cold, sterile PBS. Then, add this suspension to the second pellet and resuspend. Repeat this passage with the other two pellets.
10. Centrifuge the two 50 mL tubes at 2500× *g* for 5 min at 4 °C.
11. Gently resuspend each pellet using 2 mL of sterile and cold ultrapure water and combine the two samples together.
12. Mix the sample (total volume almost 4 mL), then split it in equal parts into four 15 mL tubes (almost 1 mL in each tube).
13. Centrifuge at 2500× *g* for 5 min at 4 °C.
14. Carefully remove the supernatant.

Note: It is crucial to remove any liquid to avoid cell pellet lysis during the freezing process. It is preferable to snap-freeze the pellets in liquid nitrogen.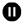 PAUSE STEP. Store cell pellets at −80 °C until further processing; otherwise, proceed with cell lysis and MNase digestion.

#### 3.3.2. Cell Lysis and MNase Digestion

Before proceeding with cell lysis and MNase digestion, make sure that all the needed buffers supplemented with protease inhibitors are prepared and cold.

Note: To inhibit protease activity, we suggest using more concentrated stocks, such as PIC 100× plus PMSF 200× or AEBSF 1000×, for smaller volumes and directly dissolving protease inhibitor tablets in a larger volume using 1 tablet for each 10 mL of volume. PMSF and AEBSF can be used as an alternative. Alternatively, to PIC 100×, it is possible to separately add other specific serine protease inhibitors, such as benzamidine (stock 0.1 M, 100×) and aprotinin (stock 2 mg/mL, 1000×), or serine, cysteine, and threonine proteases inhibitors, such as leupeptin (stock 10 mM, 1000×).
Note: Add sodium butyrate and β-glycerophosphate to the ChIP lysis buffer to inhibit deacetylases and phosphatases, respectively, and preserve histone acetylation and phosphorylation, if needed.

1. Resuspend each pellet in 400 µL of cold ChIP Lysis Buffer with 1 mM phenylmethylsulfonyl fluoride (PMSF) and 1× protease inhibitor cocktail tablet (ChIP LB+/+).
2. Transfer each suspension to a new 1.5 mL screw cap tube containing 400 μL of sterile glass beads.
3. Put the tubes into the bead-beating apparatus for high-speed agitation at 4 °C and beat 4 times for 30 s each at 6.5 m/s. Keep the cells on ice for at least 1 min between each bead beating.
4. Stab two holes at the bottom of each screw cap tube using a heated needle and collect the percolate into new tubes via centrifugation for 3 min at 2500× *g* at 4 °C.

Note: This step should remove most cell debris, but, if necessary, a further centrifugation of leachates collected in new tubes can be performed for another 2 min at 2500× g at 4 °C. The supernatant should contain the chromatin. Indeed, with 10 min of crosslinking time, the chromatin will not co-precipitate with the pellets.

5. Combine all the supernatants, collecting as much as possible (∼1600 μL); mix well; and evaluate the total volume recovered.
6. Split each sample into four 1.5 mL tubes (400 μL each) for MNase digestion reaction, keeping the remaining volume for the undigested negative control.
7. Prepare a stock of MNase Digestion Buffer (DB 3×) as indicated in Table 3, considering the column “intermediate concentration”. Consider an excess of the volume for DB 3× that will be diluted to obtain a DB 1× to dilute the MNase enzyme (step 9).
8. Add 200 μL of MNase digestion buffer every 400 μL of sample. In general, consider adding a volume of DB 3× equal to half the volume of the sample. In this way, the buffer will be diluted to reach the final concentration indicated in Table 3. Proportionally, add the DB 3× to the undigested negative control sample.
9. Dilute the MNase enzyme to a final concentration of 5 u/μL using DB 1× (obtained by diluting the DB 3× with ultrapure water), then add 10 units of MNase (i.e., 2 μL of MNase solution, 5 u/μL) to each sample to be digested.

Note: Digestion conditions should be optimized when a new stock of MNase is used. We suggest setting up the MNase digestion reaction before proceeding with the ChIP experiment, trying different amounts of enzyme and keeping the same reaction time and the same sample amount.

10. Add DB 1× to the undigested control without enzyme using an equal volume of the MNase solution used in step 9 to allow the samples to be digested.
11. Mix gently by inverting and immediately incubate in a thermal blocker at 37 °C for 15 min, 350 rpm.
12. Place the tubes on ice to stop the reaction and add 0.5 M EDTA to a final concentration of 0.020 M EDTA (i.e., 25 μL of 0.5 M EDTA to 600 μL of digested sample). Mix by inverting and briefly spin down.
13. Combine all the digested samples, then mix them by pipetting.
14. Take 40 μL of the total volume of the digested sample to process as the input sample in step 12 of Section 3.3.5. Store the input at −80 °C until processing.
15. Take 250 μL of the remaining digested sample for the MNase digestion check and proceed as described in Section 3.3.3. Store all the digested samples at −80 °C until the digestion check is complete before proceeding with chromatin immunoprecipitation.

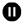
 PAUSE STEP. Store the digested samples at −80 °C until digestion is checked.
Note: If multiple chromatin immunoprecipitation reactions are planned, it is possible to split the digested samples into aquilots according to the number of IP for each round and considering 550 μL of digested sample for each IP reaction.

#### 3.3.3. MNase Digestion Quality Check

For the digestion check, proceed with the reverse crosslinking reaction of both the digested and the undigested sample aliquots, as follows:

1. Prepare the needed volume of PK buffer as described in Table 4, according to the “intermediate concentration” column.
2. Add 100 µL di PK buffer to 250 µL of sample to obtain the final concentration indicated in the last column of Table 4.
3. Add proteinase K to a final concentration of 0.4 mg/mL (for a stock of proteinase K concentrated 20 mg/mL, add a volume of enzyme equal to 1/50 of the total volume of PK buffer added to each sample). Mix gently by inverting, then briefly spin down.
4. For the reverse-crosslink reaction, incubate samples in a thermal mixer at 65 °C at 450 rpm for 12–16 h.
5. At the end of the reverse crosslinking reaction, keep the sample on ice and proceed with the DNA extraction according to this fast protocol:
  - Add 1 mL of 100% ethanol to each sample, mix well and incubate for 30 min at −80 °C.
  - Centrifuge the samples at 13,000× *g* for 45 min at 4 °C.
  - Discard the supernatant and dry out the pellet under a fume hood.
  - Resuspend the DNA in 20 μL of ddH_2_O, then add 1 µL of RNase A (20 mg/mL).
  - Mix by pipetting, then briefly spin down and incubate the reaction at 37 °C by shaking at 450 rpm for 30 min.
  - Determine the DNA concentration at Nanodrop.
  - Prepare the samples with a DNA loading dye and load the same total amount on a 1.5% agarose gel using a 100 bp ladder (EuroClone, Pero (MI), Italy).
  - Run the gel at 100 V.
  - Check the size of the fragments. MNase cuts the DNA at linker regions between nucleosomes. MNase digestion should produce fragments on an agarose gel with prominence of the mono-nucleosome band (around 147–200 bp), followed by di- and tri-nucleosomal bands. Over- or under-digestion should be minimal.

If the digestion check is successful, proceed with chromatin immunoprecipitation of the digested samples that were stored at −80 °C according to step 15 in Section 3.3.2.

#### 3.3.4. Chromatin Immunoprecipitation

1. Thaw on ice the digested sample aliquots stored at −80 °C according to step 15, Section 3.2.2, before the MNase digestion check, and bring the volumes of each IP sample to 600 µL by adding 50 µL of ChIP LB+/+ to 550 µL. Scale up the volume of ChIP LB+/+ according to the number of aliquots that will be used in the immunoprecipitation round.
2. Split 600 µL of sample for each IP reaction in a new protein low-binding tube.
3. Add the primary antibodies to the digested lysates according to their datasheets or upon protocol optimization. For the α-H7K79me1 (1 mg/mL), we used 4.8 µL for 600 µL of IP sample. For the α-H3 (1 mg/mL), we used 10 µL for 600 µL of IP sample.
4. Rotate samples on the nutator at 4 °C for 12–16 h.

Note: The addition of the primary antibodies directly to the lysates is an indirect method of immunoprecipitation which is preferred to optimize the formation of antibody–chromatin complexes; indeed, the binding between the constant region of the primary antibodies to the protein A/G coated on magnetic beads is not a limiting step. However, be aware that it is important to avoid an excess of primary antibody because any unbound antibody binds faster to the beads compared to the antibody–chromatin complexes, reducing yield.
Note: Use protein-low binding tubes for the 12–16 h incubation to minimize the antibody adhesion to the plastic tube and optimize the sample recovery.

5. After the 12–16 h incubation, start with the preparation of the beads. Place the needed volume of Protein A/G magnetic beads into a 1.5 mL protein low-binding tube. Consider 25 µL of beads for each immunoprecipitation reaction considering 10% of excess.

Note: Mix well by pipetting the beads before placing them in a new tube to ensure uniform suspension; scale up the volume according to the number of IP reactions to perform, but keep the volume of beads to a maximum of 1/3 over the wash solution volume to ensure a proper washing. If multiple tubes are used, combine all the bead aliquots at the last wash, then split 25 µL of this total volume for each IP reaction.

6. Add 900 µL of cold PBS to the beads and mix well to perform the first wash.
7. Place the tubes in a magnetic rack and let the beads collect on the side of the tube (2 min should be enough to get a limpid solution).
8. Remove the supernatant using a pipette.
9. Wash the beads successively with 900 µL of each of the buffers below as follows: twice with cold sterile PBS and four times with ChIP LB+/+. Perform each wash by rotating on the nutator for 2 min at 4 °C.

Note: Between the washes, remove the tubes from the magnetic rack and add the washing solution, then mix well by inversion and briefly spin-down; at this point, replace the tube in the magnetic rack to remove the supernatant.

10. After the last wash, add a volume of ChIP LB+/+ equal to 27.5 µL for each IP reaction (i.e., 25 µL plus 10% of excess volume) and resuspend the beads.
11. Take 25 µL of washed beads to a new protein low-binding tube.
12. Place the tubes in a magnetic stand and let the beads collect on the side of the tube.
13. Remove the supernatant using a pipette.
14. Add the antibody–chromatin complexes to the beads.
15. Rotate samples on the nutator for 1 h at 4 °C to allow the binding between the constant region of primary antibodies to the protein A/G coating of magnetic beads.

#### 3.3.5. Washes and DNA Elution

1. Place the tubes in a magnetic stand and let beads collect on the side of the tube. Remove the supernatant using a pipette. Perform each wash by rotating on the nutator for 2 min at room temperature but using cold wash solutions.
2. Wash the samples successively with 700 µL of each of the buffers below as follows:
  - Twice with cold sterile ChIP LB+/+.
  - Twice with cold sterile High Salt Wash Buffer+/+.
  - Once with cold sterile LiCl Wash Buffer+/+.
  - Twice with sterile TE Buffer.
3. Resuspend the beads in 140 µL of Elution Buffer and rotate on the nutator for 10 min at room temperature.
4. Briefly spin down, then put the tubes in the magnetic rack and transfer the supernatant into a new tube. Keep the eluted samples on ice.
5. Thaw the input samples on ice and add 100 µL of Elution Buffer to reach the same volume of immunoprecipitated eluted samples. For the next steps, treat the input as the IP reactions. Therefore, proceed with reverse crosslinking and RNase treatment.

#### 3.3.6. Reverse Crosslinking and RNase Treatment

Proceed to the reverse crosslinking and RNase treatment of the IP reaction samples, as described from step 1 to step 4 in Section 3.3.3, then proceed with DNA purification procedure described in Section 3.3.7.

Note: At this step, it is preferable to perform the DNA purification using a commercial kit with on-column DNA purification. Indeed, in this case, the purification will serve the subsequent sequencing of the samples and not the digestion control, for which it was fine to perform a faster and cheaper DNA extraction protocol as described in Section 3.3.3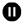 PAUSE STEP. At this point, samples can be stored at −20 °C until the DNA purification step.

#### 3.3.7. DNA Purification

1. Extract DNA with silica spin columns according to the instructions provided by the manufacturer.
2. Elute in 50 µL of RNase/DNase-free water.
3. Measure the DNA concentration at Nanodrop.
4. Proceed with the library preparation before DNA sequencing.

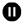
 PAUSE STEP. At this point, samples can be stored at −20 °C until the DNA library preparation and sequencing.

### 3.4. DNA Sequencing and Data Analysis

NGS of the purified DNA can be performed upon library preparation.

1. Quantify immunoprecipitated DNA with Qubit 2.0 Fluorometer.
2. Prepare the libraries using the Ovation^®^ Ultralow V2 DNA-Seq Library Preparation Kit (Tecan, Redwood City, CA, USA), strictly following the instructions provided by the manufacturer.
  - Check the quality and concentration of the final libraries both with the Qubit 2.0 Fluorometer and with the HS DNA assay Bioanalyzer.
  - Prepare the libraries for sequencing and subject them to paired-end sequencing (150 bp) on the NovaSeqX Plus platform.
3. Analyze the data. Different tools can be used for different steps of data analysis and are listed in Table 5. ChIP-seq raw FASTQ files were processed using the nf-core/chipseq v2.0.0 pipeline [28] (Zenodo doi:10.5281/zenodo.3240506) implemented in Nextflow. The pipeline ensures reproducibility by using containerized software environments provided through BioContainers, with fixed versions of the underlying bioinformatics tools defined by the pipeline release.

#### 3.4.1. Read Quality Control and Trimming

Assess the sequence quality of raw reads with FastQC v0.11.9 (Table 5) to check base quality, GC content, sequence duplication levels and over-represented sequences. Then, conduct adapter trimming using Trim Galore! V0.6.7 (FelixKrueger/TrimGalore. GitHub.) to remove sequencing adapters and low-quality bases.

#### 3.4.2. Alignment and Post-Processing

Align reads to the reference genome (using *Saccharomyces_cerevisiae*. R64 fasta file and *Saccharomyces_cerevisiae*.R64-1-1.115.gtf for gene annotation) with BWA v0.7.17 [30] aligner. Following alignment, coordinate-sorted BAM files are generated.

Identify technical duplicates and mark them using Picard v2.23.1 (Table 5). Estimate library complexity metrics using Preseq v3.1.2 (Table 5) to check if the libraries have low complexity, which is associated with a high PCR-duplicated library. Remove unmapped reads, reads with an insert size > 2 kb, non-primary alignments, and reads with low mapping quality (MAPQ < 6) using SAMTools v1.15.1 [31].

#### 3.4.3. Signal Generation and Peak Calling

Generate normalized BigWig tracks (reads per million mapped) using BEDTools v2.29.2 [32] and the UCSC tool bedGraphToBigWig for downstream visualization in Integrative Genomics Viewer (IGV). Peaks can be analyzed using MACS2 v2.2.7.1 [33] for narrow or broad peaks as appropriate. A consensus peak set across all samples can be generated using BEDTools, and read counts within peaks extracted using feature Counts v1.6.4 [37].

Perform downstream sample clustering with a principal component analysis in R v4.3.1 using the DESeq2 v1.28.0 package [35].

Compile the multi-omics summary and QC report using MultiQC v1.13 [36].

### 3.5. Troubleshooting

The MNase-ChIP-seq workflow involves multiple steps, several of which require adjustment depending on the available reagents and the specific experimental goals. In this section, we provide a list of common issues that may arise during the procedure, together with potential solutions (Table 6).

## 4. Expected Results

We validated the MNase-ChIP-seq protocol by analyzing the methylation of lysine 79 in histone H3 (H3K79) in the surroundings of the unrepairable HO-induced DSB in asynchronous cells. Mono-, di-, and tri-methylation of H3K79 are induced by the evolutionarily conserved Dot1 (DOT1L in mammals) methyltransferase [38,39] and are known to be involved in DSB signaling and repair both in yeast and in mammals [8,40,41]. Specifically, we evaluated the enrichment of the mono-methylated variant of H3K79 (H3K79me1) at the centromere-proximal region to the HO-cut site on chromosome III.

**Figure 5:**
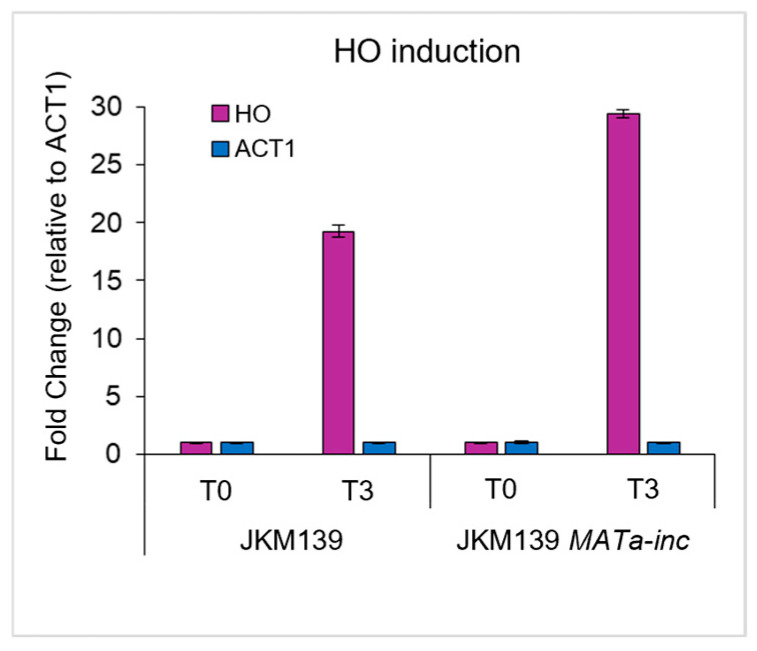
HO induction by galactose. Galactose was added to exponentially growing JKM139 and JKM139 *MATa-inc* cell cultures. RNA was extracted from samples taken before (T0) and three hours after galactose addition (T3) and subjected to RT-qPCR with primer pairs targeting the *HO* gene and the *ACT1* control.

Galactose was added to exponentially growing JKM139 and JKM139 *MATa-inc* cells at time 0 to induce HO expression. Samples were collected at time 0 and 3 h after galactose addition to evaluate HO induction and the efficiency of DSB formation and to perform MNase-ChIP-seq with antibodies against both the H3K79me1 variant and total H3. HO expression increased 20–30 folds after 3 h in galactose-containing medium compared to the uninduced condition in both JKM139 and JKM139 *MATa-inc* cells, as revealed by RT-qPCR analyses (Figure 5). Furthermore, we determined an HO-cut efficiency of 98% at the *MATa* locus in JKM139 cells 3 h after galactose addition and around 0% in the JKM139 *MATa-inc* control strain by qPCR analyses. These results confirmed that a DSB was efficiently induced at the *MATa* locus of JKM139, while the same locus remains intact in the control strain, despite the HO induction.

**Figure 6:**
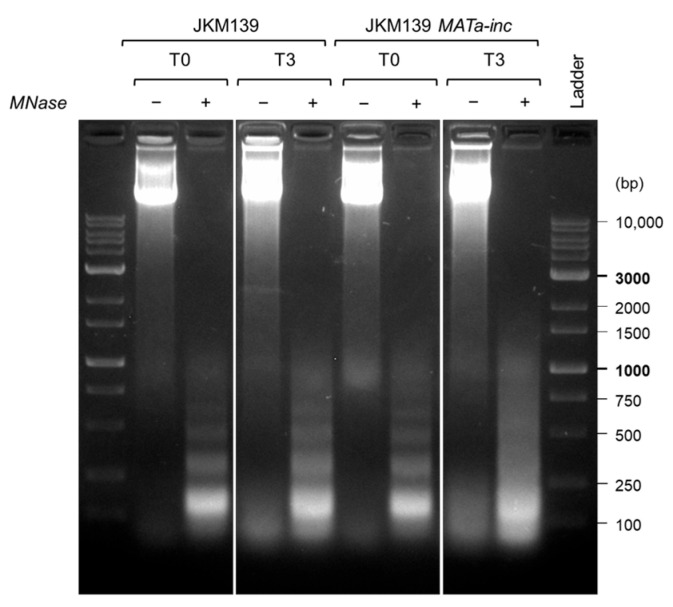
MNase digestion check. Micrococcal nuclease was added to cell lysates, followed by incubation at 37 °C for 15 min. MNase-treated (+) and untreated (−) samples were analyzed by electrophoresis in an agarose gel. T0 and T3 indicate time zero (uninduced) and three hours later galactose induction, respectively. Ladder: SHARPMASS 1 Kb plus, ready-to-load 1 Kb ladder (Euroclone, product code: EMR81,600).

We therefore proceeded with crosslinking, cell lysis and chromatin digestion with MNase for 15 min at 37 °C. This treatment led to the formation of chromatin fragments mainly containing mono-nucleosomes (approximately 150 bp), with smaller amounts of di-nucleosomes (approximately 250 bp) and few larger fragments (Figure 6). As MNase treatments generated similar digestion patterns and mainly mono-nucleosomes in all the samples, we proceeded with the ChIP-seq steps.

**Figure 7:**
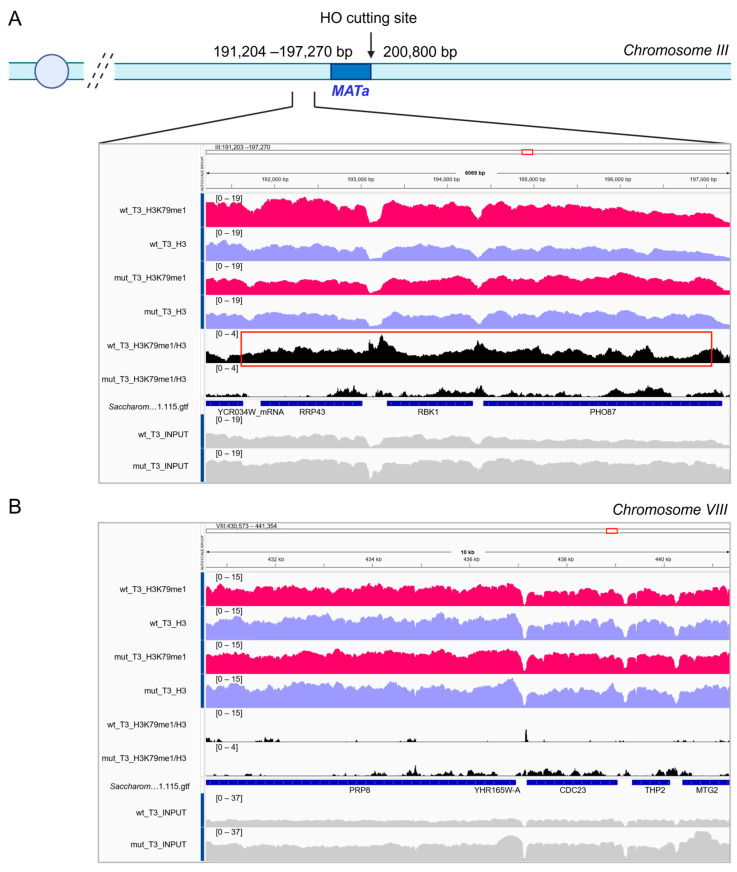
H3K79me1 is enriched at the HO-induced DSB. (**A**). Representative IGV browser tracks of H3K79me1 (pink tracks) and H3 (purple tracks) peaks in the JKM139 (wt) and JKM139 *MATa-inc* (mut) strains in the centromere-proximal (chromosome III, 191,204–197,270 bp) region of the DNA double-strand break (chromosome III, 200,850 bp) at T3 (three hours) upon galactose induction. Schematic representation of the right arm of chromosome III is shown on top. The *MATa* locus and the coordinates of both the HO cutting site and the regions analyzed with the IGV tracts are indicated. (**B**). Representative IGV browser tracks in the unrelated control region (chromosome VIII, 430,573–197,270 bp). All IGV tracks in a given comparison have the same scaling factor for the y-axis, as indicated in the upper left-hand region of each track. The RefSeq gene map is presented in blue at the bottom of the tracks panel, showing the overall reference genome structure. The relative ratio H3K79me1/H3 is shown in the black tracks for both strains.

MNase-digested samples were immunoprecipitated with antibodies against either the H3K79me1 variant and total H3, followed by DNA purification and sequencing (see Figure 2 and Figure 3). Then, we verified that DSB formation leads to an accumulation of H3K79me1 by analyzing the enrichment of HO-cut centromere-proximal reads (sequence spanning from 191,204 to 197,270 of chromosome III) in H3K79me1 and H3 immunoprecipitated samples three hours after the HO-cut induction (Figure 7). As nucleosomes are known to be evicted at DSB ends while chromatin modifications expand for several kilobases in the surroundings of DSBs [8], we analyzed a region that is not immediately adjacent to the DSB, but comprises ~6500 bp at almost 3 kb from the left side of the HO cutting site (Figure 7A). To confirm that H3K79me1 enrichment is specific to the region surrounding the DSB, we performed the same analysis at an unrelated locus on chromosome VIII (Figure 7B). As expected, the relative H3K79me1/H3 ratio increased in the wild-type JKM139 strain compared to the mutant JKM139 *MATa-inc* strain at chromosome III three hours after HO induction (Figure 7A, black tracts). Conversely, the H3K79me1/H3 ratio at the control region on chromosome VIII was comparable in JKM139 and JKM139 *MATa-inc* strains (Figure 7B). These findings indicate that DSB formation causes the enrichment of the H3K79me1 variant for at least 8 kb in the left arm of the DSB.

## 5. Discussion

In this work, we present a robust MNase-ChIP-seq protocol designed to map genome-wide histone PTMs in *Saccharomyces cerevisiae* following the induction of a single, irreparable DSB. The system relies on the inducible expression of the HO endonuclease, which efficiently generates a persistent DSB at a defined genomic locus. The stability of this break provides a favorable context to monitor the accumulation, removal, and temporal evolution of histone PTMs, which are normally highly dynamic during DSB repair. By combining MNase digestion, ChIP, and NGS techniques, this protocol enables a detailed analysis of histone PTMs surrounding a DSB. Importantly, it can be readily integrated with complementary methods that monitor ssDNA generation or recruitment of DNA repair and checkpoint proteins at the same site, thus allowing direct correlation of histone PTMs dynamics with DSB signaling, processing and eventually repair.

Beyond the analysis of histone PTMs, the flexibility of this protocol makes it suitable for studying the binding of non-histone chromatin-associated proteins, provided that appropriate antibodies are available. By selecting the most suitable chromatin fragmentation strategy—MNase digestion for nucleosome-resolved profiles or sonication for more heterogeneous chromatin contexts—the workflow can be adapted to investigate the recruitment of DNA repair proteins, transcription factors, and chromatin remodelers either at the DSB site or genome-wide. Moreover, since many proteins involved in the DNA damage response undergo PTMs themselves, the use of antibodies recognizing specific modified forms of these proteins can reveal the presence and kinetics of these modifications at or around the break, offering additional insight into the regulation of the DNA damage response. The protocol is highly versatile and can be adapted to different yeast backgrounds, to alternative genomic loci, or to breaks generated by other nucleases, including repair-competent DSBs. It supports comparative studies across mutant strains or stress conditions and can be expanded by increasing the number of antibodies or time points analyzed, providing a dynamic view of histone PTMs appearance and spreading during the DNA damage response. Altogether, this system provides a powerful framework for dissecting chromatin remodeling at DNA breaks and linking histone PTMs with DSB processing and checkpoint control.

## 6. Reagents Setup

Prepare all solutions using filtered and deionized ultrapure water (ddH2O; resistivity 18.2 MΩ·cm at 25 °C) and analytical-grade reagents. Prepare and store all solutions at room temperature (unless indicated otherwise).

### 6.1. Reagents for Yeast Cells Growth and DSB Induction

- 30% D-(+)-Raffinose 30%: Dissolve D−(+)−raffinose pentahydrate in ddH_2_O. Autoclave or sterilize by filtration.
- 30% D-(+)-Galactose: Dissolve D−(+)−galactose ≥ 99.0% in ddH_2_O. Sterilize by filtration. Do not autoclave. Galactose should not be autoclaved because it isomerizes at elevated temperatures.
- YEPD medium: 2% Bacto peptone, 1% yeast extract, 2% D_(+)_glucose monohydrate, 0.005% adenine hemisulfate salt. Dissolve in ddH_2_O. Autoclave.
- YEPR medium: dissolve 2% Bacto peptone, 1% yeast extract, 0.005% adenine hemisulfate salt in ddH_2_O. Autoclave. Add sterilized raffinose from 30% solution to 2%.

### 6.2. Reagents for Evaluation of HO Expression and DSB Formation

- Lysis buffer: 0.5 M NaCl, 0.2 M Tris-HCl pH 7.5, 10 mM EDTA, 1% SDS.
- Ice-cold ethanol: 99% and 70%. Prepare a 70% ethanol solution by diluting 99% ethanol in ddH_2_O. Store at −20 °C- aliquots of both 70% and 99% ethanol in glass bottles.
- 5× TBE buffer: Prepare a 5× stock solution with 54 g of Trizma Base (Tris base), 27.5 g of boric acid, and 20 mL of 0.5 M EDTA (pH 8.0). Add 800 mL of distilled water to dissolve using a magnetic stirrer. Allow to stir until the solution becomes clear. Adjust the volume with distilled water up to 1 L. The pH of the concentrated stock buffer should be ~8.3. Autoclave the solution. Store at room temperature.
- Spheroplasting solution: 0.9 M sorbitol; 0.1 M EDTA, pH 7.5.
- Zymolyase solution: Dissolve 2 mg/mL Zymolyase 20 T from *Arthrobacter luteus* (Nacalai Tesque) in spheroplasting solution + 14 mM β−mercaptoethanol.
- 1× TE: 10 mM Tris–HCl, pH 7.5; 1 mM EDTA, pH 7.4. Autoclave.
- Lysis solution: 2.2% sodium dodecyl sulfate (SDS); 278 mM EDTA, pH 8.5; 445 mM Tris-base. Prepare the lysis solution just before adding it to the samples by mixing the appropriate amounts of 10% SDS; 0.5 M EDTA, pH 8.5; 2 M Tris-base stock solutions in a 15 mL tube.
- 5 M potassium acetate: Dissolve 5 M potassium acetate in ddH_2_O. Autoclave.
- RNase solution (10 mg/mL): Dilute the RNase A solution (20 mg/mL) by adding an equal volume of DNase–free water.

### 6.3. Reagents for Chromatin Immunoprecipitation

- HEPES 1 M pH 7.5: Dissolve 238.3 g of HEPES in 800 mL of ddH_2_O. Adjust the solution to pH 7.5 with sodium hydroxide (10 N). Add ddH_2_O until the volume is 1 L. Sterilize by filtration. Store at 4 °C.
- PMSF 200× (0.2 M): Dissolve PMSF in ethanol. Store at −20 °C.
- AEBSF 1 M: Dissolve in ddH_2_O. Store aliquots at −20 °C. Stock solutions are stable for up to 6 months at −20 °C.
- Benzamidine 0.1 M: Dissolve in ddH_2_O. Store aliquots at −20 °C. Stock solutions are stable for up to 6 months at −20 °C.
- Aprotinin (2 mg/mL): Dissolve in ddH_2_O. Store aliquots at −20 °C. Stock solutions are stable for up to 6 months at −20 °C. Repeated freeze–thaw cycles should be avoided.
- Leupeptin 0.01 M: Dissolve in ddH_2_O. Store aliquots at −20 °C. Stock solutions are stable for up to 6 months at −20 °C.
- ChIP Lysis Buffer (ChIP LB): 0.05 M HEPES pH 75, 0.14 M NaCl, 0.001 M EDTA, 0.1% NadEox, 1% IGEPAL. Store the solution at 4 °C.
- ChIP LB+/+: Prepare fresh starting from cold ChIP LB and adding the protease inhibitors and other inhibitors as needed.
- 0.5 M CaCl_2_: Dissolve 11 g of CaCl_2_•6H_2_O in a final volume of 20 mL of ddH_2_O. Sterilize the solution by passing it through a 0.22 µm filter. Store at 4 °C.
- (3×) MNase digestion buffer (DB): Tris-HCl 0.15 M pH 7.5, CaCl_2_ 0.018 M, PIC (3×), ddH_2_O. Prepare fresh MNase digestion buffer (3×) as follows: for 1.5 mL of total volume, mix 225 µL Tris-HCl 1 M, pH 7.5, 54 µL 0.5 M CaCl_2_, 15 µL PIC 100× and 1206 µL ddH_2_O. Keep on ice until use.
- (1×) MNase digestion buffer (DB): Tris-HCl 0.05 M pH 7.5, CaCl_2_ 0.006 M, PIC (1×), ddH_2_O. Prepare fresh by diluting the (3×) MNase digestion buffer with ddH2O. To obtain 1.5 mL of DB (1×) mix 0.5 mL of DB (3×) and 1 mL of ddH_2_O. Keep on ice until use.
- (3.5×) PK buffer: Tris-HCl 0.175 M pH 7.5, NaCl 0.014 M, CaCl_2_. 0.0105 M, SDS 3.5%. To get 1 mL of PK buffer, mix 175 µL Tris-HCl 1 M pH 7.5, 2.8 µL NaCl 5 M, 21 µL CaCl_2_, 451.2 µL ddH_2_O, and finally 350 µL SDS 10%.
- High-Salt Wash Buffer +/+: 0.05 M Tris-HCl pH 7.5, 0.5 M NaCl, 0.001 M EDTA, 1% IGEPAL, PIC 1×. Once freshly prepared, keep on ice until use.
- LiCl Wash Buffer +/+: 0.01 M Tris-HCl pH 7.5, 0.25 M LiCl, 0.001 M EDTA, 1% IGEPAL, 1% NadEOX, PIC 1×. Once freshly prepared, keep on ice until use.
- TE Buffer: 1 M Tris-HCl pH 7.5, 0.5 M EDTA pH 8.
- Elution Buffer: 0.001 M NaHCO_3_ + 1% SDS. Prepare a stock solution of NaHCO_3_ 1 M by dissolving 8.4 g of NaHCO_3_ in 0.08 L of ddH_2_O. Once dissolved, bring the volume to 0.1 L with ddH_2_O. Filter to sterilize the solution. Store at room temperature.

## Figures and Tables

**Table 1:** List of primer pairs for qPCR to evaluate the HO expression upon induction of the *GAL1* promoter.

Gene	Primer	Sequence (5′–3′)
HO	Sc_HO_Fw	AGTCAGGGTCCCTACCAAAC
	Sc_HO_Rev	GCGGCAAACTCACCTTCAAC
ACT1	Sc_ACT1_Fw	TCGCCTTGGACTTCGAACAA
	Sc_ACT1_Rev	TACCGGCAGATTCCAAACCC
GAL1 ^1^	Sc_GAL1_Fw	GCAGTTGAAGGCTACTCCGT
	Sc_GAL1_Rev	ACCGTACGTGGCAGCTAAAA

^1^ The GAL1 primer pair can be used as a positive control of galactose induction (optional step).

**Table 2:** List of primer pairs for qPCR to evaluate the HO cut efficiency upon induction of HO expression.

Gene	Primer	Sequence (5′–3′)
HO	Sc_HO+	GTGGCATTACTCCACTTCAA
	Sc_HO-	TCACCACGTACTTCAGCATA
KCC4	Sc_KCC4+	TCGTATCAGGTCTGCCCTATGAA
	Sc_KCC4-	CTCTGGAAATTTCGGTGTCATTG

**Table 3:** Set up of MNase digestion buffer (DB).

Reagent	Intermediate Concentration (DB 3×)	Final Concentration (DB 1×)
Tris-HCl 1 M, pH 7.5	0.15 M	0.05 M
CaCl_2_ 0.5 M	0.018 M	0.006 M
PIC 100×	3×	1
H_2_O	Up to tot volume	

Note: Use freshly prepared and no more than two-month-old CaCl_2_; otherwise, the efficiency of MNase digestion may be affected.

**Table 4:** Setup of PK reaction buffer.

Reagent	Intermediate Concentration (PK 3.5×)	Final Concentration (PK 1×)
Tris-HCl 1 M, pH 7.5	0.175 M	0.05 M
NaCl 5 M	0.014 M	0.004 M
CaCl_2_ 0.5 M	0.0105 M	0.003 M
10% SDS *	3.5%	1%
H_2_O	Up to total volume	

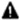
 CRITICAL STEP: * Add last to avoid precipitation.

**Table 5:** List of tools and related references for the data analysis.

Tool	Version	Reference or Link
Nextflow	21.10.3+	[29]
FastQC	0.11.9	*https://www.scirp.org/reference/referencespapers?referenceid=4024153*
Trim Galore!	0.6.7	*https://github.com/FelixKrueger/TrimGalore*
BWA	0.7.17	[30]
SAMtools	1.15.1	[31]
Picard	2.27.4	*https://broadinstitute.github.io/picard*
Preseq	3.1.2	*https://smithlabresearch.org/software/preseq/*
BEDTools	2.30.0	[32]
MACS2	2.2.7.1	[33]
deepTools2	3.5.1	[34]
DESeq2	1.28.1	[35]
MultiQC	1.13	[36]

**Table 6:** Troubleshooting for MNase-ChIP-seq and potential solutions.

Issue	Observation	Potential Explanation	Potential Solutions
Insufficient cell lysis	Low chromatin extraction; reduced immunoprecipitation efficiency	Incomplete mechanical or enzymatic cell lysis prevents chromatin release	Verify cell lysis microscopically; ensure > 90% of cells are lysed (keep a small portion of not lysed cells as negative control). Optimize bead-beating duration or enzymatic digestion conditions according to the strain and growth phase
Insufficient MNase digestion	Predominantly long chromatin fragments, elevated background signals and reduced resolution.	MNase activity varies between batches. Suboptimal enzyme concentration or incubation time	Empirically determine the appropriate MNase concentration and digestion time for each batch. Adjust condition to obtain predominantly mononucleosomal or dinucleosomal fragments. Test digestion on a small aliquot before proceeding with immunoprecipitation
Insufficient HO induction	Low or undetectable DSB levels; poor accumulation of DSB-dependent histone PTMs	Inefficient induction of HO endonuclease expression or defects in HO cutting at the target site	Verify HO mRNA and/or protein expression after galactose induction. Confirm cutting efficiency at the target site (Section 3.2.2). Optimize induction time and galactose concentration
Antibody specificity and high background	High background signal; ambiguous PTMs enrichment profiles	Antibodies cross-reactivity; poor specificity; Nonspecific chromatin binding to beads	Validate the ChIP efficiency for each antibody by qPCR at genomic regions known to be enriched for the PTM of interest. Include IgG antibodies as a control for background subtraction

## Data Availability

The original contributions presented in this study are included in the article. Further inquiries can be directed to the corresponding author.

## Abbreviations

The following abbreviations are used in this manuscript:

DSB	Double-strand break.
NHEJ	Non-homologous end joining.
HR	Homologous recombination.
PTMs	Post-translational modifications.
MS	Mass spectrometry.
ChIP	Chromatin immunoprecipitation.
ChIP-seq	Chromatin immunoprecipitation followed by sequencing.
CUT&RUN	Cleavage Under Targets and Release Using Nuclease.
MNase	Micrococcal nuclease.
PK	Proteinase K.
LB	Lysis Buffer.
DB	Digestion Buffer.
